# A multicenter randomized controlled trial protocol to evaluate the effectiveness of an educational intervention on fertility knowledge, intention and behavior among Iranian new couples

**DOI:** 10.1186/s12889-020-10029-4

**Published:** 2020-12-17

**Authors:** Maryam Gharacheh, Farideh Khalajabadi Farahani, Mojgan Mirghafourvand, Leila Janani, Fahimeh Ranjbar

**Affiliations:** 1 Nursing Care Research Center, School of Nursing and Midwifery, Iran University of Medical Sciences, Tehran, Iran; 2 Department of Population & Health, National Population Studies & Comprehensive Management Institute, Tehran, Iran; 3 Midwifery Department, Social Determinants of Health Research Center, Tabriz University of Medical Sciences, Tabriz, Iran; 4 Preventive Medicine and Public Health Research Center, Psychosocial Health Research Institute (PHRI) & Department of Biostatistics, School of Public Health, Iran University of Medical Sciences, Tehran, Iran

**Keywords:** Fertility knowledge, Childbearing intention, Behavior, New couple, Iran

## Abstract

**Background:**

Recent evidence shows that men and women have inadequate fertility knowledge which may negatively affect their childbearing decisions in future. Given the fact that decision making for fertility needs accurate information, targeted educational interventions especially through media are needed to improve knowledge regarding the best age of fertility, factors affecting fertility potential and fertility options available for sub-fertile couples. Aim of the study is to evaluate whether a fertility educational program can be effective in increasing fertility knowledge, childbearing intention and the planned pregnancy rate among couples referring to premarital counselling centers.

**Methods:**

This study is a parallel randomised clinical trial with pre-test/post-test design. We will recruit 1240 marrying couples referring for compulsory premarital counselling in public health centers through stratified sampling in five metropolitan cities of Iran. The intervention group will receive both the typical premarital counselling training and a fertility knowledge package containing verbal and virtual educational package at five time episodes (one verbal session and four virtual sessions) within 4 weeks. The primary outcomes are fertility knowledge, childbearing intention and the first planned pregnancy rate (positive pregnancy test) and the secondary outcomes include contraception method use, miscarriage and unplanned pregnancy. Participants will respond to a self-administered demographic/reproductive characteristics questionnaire, the Cardiff Fertility Knowledge Scale (CFKS) and the childbearing intention questionnaire.

Data will be collected through online questionnaires at baseline and 3, 12 and 18 months after the intervention. Data will be analyzed using Chi-square or Fisher-exact test for categorical variables, Independent sample t-test for normally distributed quantitative variables and Mann–Whitney U test for non-normally distributed quantitative variables. To compare the outcomes between the two groups over the time, repeated measures ANOVA will be used. We hypothesize that the positive impact of increasing the fertility knowledge is the reduced involuntarily childlessness.

**Discussion:**

The findings are proposed to inform government policies and public education strategies aiming at supporting childbearing among young couples who postpone their first pregnancy while they might not have any important social and economic obstacles.

**Trial registration:**

This study was approved by Iranian Registry of Clinical Trials (IRCT), Number: IRCT20201005048925N1, Date of registration: 2020-10-12.

## Background

Recent evidence shows that men and women have inadequate fertility knowledge and awareness which may negatively affect their childbearing decisions in future [1–5]. For instance, some people underestimate the effect of age on fertility potential and overestimate the success rates of assisted reproduction [3, 6, 7]. They are unaware of consequences of postponed parenthood that may lead to a higher rate of infertility and maternal and child health risks with higher female and male age at fertility [8–10]. Therefore, there is a serious need for public education about age-related fertility declines and the availability, and limitations of assisted reproduction technologies [11, 12]. Hence, educational interventions are important to promote fertility knowledge and ensuring couples have a realistic perspective of fertility treatment [13].

Most of the people obtain fertility knowledge from the mass media or internet. Given the fact that decision making for fertility needs accurate information [5], targeted educational interventions specially through media are needed to improve knowledge regarding the best age of fertility, factors affecting fertility potential and fertility options available for sub-fertile couples [4, 14]. The majority of studies on fertility knowledge are cross-sectional and have studied women [14], university students [15–20] and/or people from high-income countries [4, 5, 10, 21] that making it difficult to generalize findings to new couples in developing countries.

In Iran, the fertility rates have dramatically declined over the past three decades, and the increased marriage age, delayed childbirth, significant rate of infertility (20.2) and a recent rise in voluntary childlessness require serious attention [22–24]. The total fertility rate in Iran has decreased in recent years and it fell into 2.2 in 2000,1.9 in 2006 and 1.7–1.8 in 2019, respectively [25]. Below replacement fertility has created some concerns among policymakers and scholars in Iran and has led to the announcement of pronatalist population policies in 2014 by the Supreme Leader [26]. Studies have also shown that fertility ideals are greater than fertility intention and behavior (well about 2 children) among the new couples in Tehran [27–29]. Therefore, any attempt to help couples to achieve their fertility ideals by removing obstacles of childbearing will be in line of new population policies of Iran.

Studies show that many social, economic and individual factors contribute to the lower fertility behavior among new generations. For instance, individual factors, women’s higher education and employment have been shown to be associated with lower fertility rate. Apart from these, poor fertility knowledge and misconceptions might be responsible for delay in childbearing among young couples [5, 30]. Evidence shows that people have little information about factors reducing their fertility chance [1, 6, 13]. A study in Iran also showed that 56% of Iranian couples did not have correct fertility information. Therefore, comprehensive educational interventions on fertility and factors reducing the fertility potential might be an effective strategy to help men and women to achieve their fertility goals before it gets late or they face infertility [31]. However, there is no evidence in Iran that how effective such interventions are in improving fertility knowledge and intention.

Fertility changes in Iran have been well monitored by demographers in recent years, and various individual and socio-economic factors have been considered to be responsible for fertility rate. Some new population policies have been introduced to encourage childbearing such as increased parental leave for a childbirth, while little has been done with regard to improvement of fertility knowledge. Considering the high rate of primary infertility (20.2%) [22], and a growing number of women electing to postpone childbearing in Iran [10, 23], it is important to assess fertility knowledge of new couples and also the effectiveness of an educational intervention based on fertility knowledge in their reproductive intention and behaviors. Evaluating the number of planned pregnancy (as a long term goal) in our study will demonstrate to what extent fertility knowledge can be translated to childbearing behavior.

## Methods/design

### Study aim

To evaluate the effectiveness of an educational intervention program on fertility knowledge, childbearing intention and planned pregnancy rate.

### Trial design

This study is a parallel randomised clinical trial with pre-test/post-test design among 1240 couples referring to premarital counselling centres. The flow diagram of the study design is reported in Table 1. We will also conduct a pilot survey on 30 couples to ensure the feasibility of the study, the appropriateness of the educational package and possible obstacles and problems and appropriateness of the study instrument.

**Table 1:** Flow diagram of the study design

	STUDY PERIOD				
	Pre-intervention		Post- intervention		
**TIMEPOINT**	Enrolment	Allocation	3th month	12th month	18th month
**Enrolment**					
Eligibility screening	**×**				
Informed consent	**×**				
Randomization		**×**			
**Interventions**					
Intervention (fertility knowledge training)			**×**	**×**	**×**
Control (typical premarital counselling)			**×**	**×**	**×**
**Assessments**					
**Primary outcomes**					
fertility knowledge		**×**	**×**	**×**	**×**
childbearing intention		**×**	**×**	**×**	**×**
first planned pregnancy		**×**	**×**	**×**	**×**
**Secondary outcomes**					
contraception method use		**×**	**×**	**×**	**×**
miscarriage		**×**	**×**	**×**	**×**
unplanned pregnancy		**×**	**×**	**×**	**×**

### Setting and participants

This study will be conducted on couples referring for compulsory pre-marriage training in public health centers in five metropolitan cities of Iran with a diverse geographical distribution (Tehran, Mashhad, Ahvaz, Tabriz and Shiraz) to enhance the generalizability of the findings. We plan to recruit new couples (both men and women) referring for compulsory premarital counselling.

### Inclusion criteria

Marrying couples, having an Iranian nationality with minimum basic literacy, women aging from 18 to 35 years, men aging 18–45, with no previous marriage, referring to premarital counselling centers, and are about to live together in their new home (under one roof) within 1 month, will be included in the study. We will select a low-risk group of women for pregnancy according to women’s age.

An increasing number of women delay having children until after 35 years. Fertility clearly declines with advancing age, especially after the mid-30s and women are at greater risk of pregnancy complications. Therefore, we will include only women aged 18–35 as these women are making choices that may affect their intentions regarding the timing of childbearing (e.g., following education or career) and usually postpone childbearing decisions [32]. Semen parameters in men also decline detectably after 35 years of age but male fertility does not appear to be affected before 50 years old [33].

### Exclusion criteria

Medical students or staff, participants who have any known chronic diseases and marrying couples who did not start living together will be excluded.

### Interventions

The intervention group will receive both the typical premarital counselling and a fertility knowledge training containing verbal and virtual educational program at five time episodes (one verbal session and four virtual sessions) in a package. We will implement the educational programs during 4 weeks. The intervention includes a 30-min lecture, documentary videos, text messages and short films. The control group will receive only typical premarital counselling. The content of educational or counselling program will be based on the most updated literature on fertility knowledge.

The educational program was developed based on new guidelines of American Society for Reproductive Medicine (ASRM), European Society of Human Reproduction and Embryology (ESHRE) and Iran’s Ministry of Health and Medical Education (MOHME).

The fertility knowledge package will consist of information on fertility rates, infertility rates, risks of delay in childbearing, safe waiting period for parenting, Impact of age on female fertility, limited fertility period for women, fertility window and how to optimize fertility, impact of weight and lifestyle on fertility, definition of infertility, infertility risk factors, impact of sexually transmitted diseases (STDs) on fertility, the need for earlier evaluation and treatment of infertility, success rate and financial costs of infertility treatments, assisted conception and fertility preservation. In order to incorporate multidisciplinary views in this study, specialists from different relevant disciplines will be invited to provide their views on different stages of the study including the suitability of the educational content. A group of experts composed of midwife, reproductive health professional, gynecologist (infertility fellowship), medical sociologist, health psychologist and health education will evaluate and confirm the scientific validity of the intervention package. The content of educational program will be also assessed in the pilot phase.

Instructors will be trained by the principal investigator before the intervention and their questions will be answered. The educational content will be provided to them as a booklet for future reading. Further, they will be asked to record the educational counselling session as an audio file for evaluation by the principal investigator. In order to increase the quality of the training program, some features will be considered, such as having a long training period (1 month), using the power of media, having intermittent training with reminders and repeating in regular basis, and in this way the triangulation will enhance the quality of the counselling program.

### Outcomes

The primary outcomes are fertility knowledge, childbearing intention and the first planned pregnancy rate (positive pregnancy test).

The secondary outcomes include the contraception method use, miscarriage and unplanned pregnancy.

### Data collection

Data will be collected by questionnaires at baseline and 3, 12 and 18 months after the intervention in each selected city (Table 1). The post-test questionnaires will be completed by the participants through online forms. The participants’ phone number, email address and social network’s ID (WhatsApp) will be asked in order to send the post-test questionnaire links and some virtual educational materials.

In each city, two research collaborators will cooperate with the principal investigator. They will be introduced by the Ministry of Health and Medical Education, associated office for premarital counselling courses in all provinces. We will have a training session for these research staff and they will be justified about the study aims and procedure of data collection as well as educational intervention. They need to be in close contract with the principal investigator.

To minimize loss to follow up, we will provide an incentive to the eligible participants (A gift voucher for free sexual and reproductive health counselling) on the completion of the survey which may increase response rates. Four reminder emails or text messages will send out to non-respondents at one, two, three, and 4 weeks; a fifth telephone reminder was also undertaken.

Since a recent study in Iran showed an average interval between marriage and first pregnancy as about 15.4 ± 0.2 months in Iran [24], we are planning to follow the participants for occurrence of planned pregnancy about 18 months after the intervention.

### The study instrument

Participants will respond to a self-administered demographic characteristics questionnaire, the Cardiff Fertility Knowledge Scale (CFKS), the childbearing intention questionnaire and some questions about their pregnancy (planned/unplanned pregnancy).

#### Demographic characteristics questionnaire

Participants will complete the socio-demographic characteristics form including age, education level, income, occupation, ethnicity, gender and geographic area (province and city).

#### Cardiff fertility knowledge scale (CFKS)

The Persian version of the Cardiff Fertility Knowledge Scale (CFKS) will be used to assess the fertility knowledge. The CFKS consists of 13 items measuring knowledge about facts, risks and myths of fertility. Participants will respond to all items as true, false or do not know. A correct answer is assigned one point and an incorrect or ‘do not know’ answer is assigned zero point. Scores are reported as the percent correct score (0–100%). The Cronbach’s alpha coefficient of the CFKS was 0.79 and satisfactory for most countries [13].

#### Childbearing intention questionnaire

Parts of a questionnaire developed by Lampic et al. (2006) will be used in this study as the basis for the data collection regarding childbearing intentions that has satisfactory face validity and reliability and has previously been used by Lampic et al. (2006), Peterson et al. (2012) and Chan et al. (2014) in their studies of Swedish, American and Hong Kong university students [6, 16, 19]. The questionnaire contains five parts: (i) perceived knowledge of fertility issues (two items) asking about the perceived level of fertility-related knowledge; (ii) intention and potential obstacles of childbearing (seven items) including yes–no questions and open-response questions that aim to understand the ones’ intention to have children, as well as perceived obstacles; (iii) awareness of fertility issues (eight items) consisting of open-response items concerning changes in female fertility with age, and likelihood of pregnancy and infertility; (iv) importance of childbearing and intended behavior in the case of infertility (four items) containing four 0- to 10-point response scales assessing the perceived importance of childbearing and the preferred course of action in the case of infertility, and (v) conditions for parenthood (13 items) such as emotional readiness and financial stability that may be important in people’s decision to have children [16].

### Validity and reliability

After requesting the permission of the original authors, the instrument will be translated by a bilingual researcher into Persian, and the back-translation will be done independently by another bilingual researcher (in the field of reproductive health) and a native English speaker. An expert panel will be held and the questionnaire will be restructured based on their remarks and comments. Some of the questions may be revised based on cultural issues and the inclusion criteria in the current study. To determine the validity of the instrument, it will be provided to several experts in the field of reproductive health and their views will be collected and the instrument will be amended accordingly. Test-retest and Cronbach’s alpha coefficient will be used to estimate the reliability of the instrument.

### Sample size

According to our primary outcomes including fertility knowledge and intention toward childbearing that are important to be measured 3 months after the intervention, we considered a small standardized Cohen effect size (d = 0.2) with a type I error of 5% (α = 0.05) and a type II error of 10% (β = 0.1; power = 90%) and sample size obtained 527 in each group and considering 15% of loss to follow up, the sample size was calculated 620 couples in each group using the G-Power software. Considering the design effect equals to 1.5, final size was calculated 1240 couples in total (620 couples in each group). In each city around 200 couples (100 in each group) and from Tehran about 440 couples (220 in each group) will be recruited.

It should be noted that in order to compare quantitative outcomes between groups over time (baseline, 3, 12 and 18 month), the sample size obtained 660 couples in total (330 in each group) based on repeated measures ANOVA. So, the initial obtained sample size (620 couples in each group) will have the appropriate power for this analysis.

### Randomization

Samples will be selected through stratified sampling and random samples are then selected from each stratum. The strata consist of five cities, and separate randomization lists will be prepared for each city using a block randomization.

Due to the limited number of main public centers providing compulsory premarital counselling in each city (3 centers in Tehran, 2 centers in in Ahvaz, 1 center in Shiraz, 2 centers in Tabriz and 3 centers in Mashhad), and other concerns such as selection bias, and generalizability (according to potential confounder of socioeconomic status), the cluster randomization based on the centers is not possible. So, we will use central randomization that is stratified to the cities.

The PI will randomly assign the couples into two groups in each city by a computer-generated random sequence. For randomization, the permuted block randomization will be used (block size = 4). According to the sample size of 440 identified, 110 blocks will be produced using the online site (www.sealedenvelope. com) for Tehran. According to the sample size of 200 identified, 50 blocks will be produced using the online site (www.sealedenvelope. com) for other cities.

Allocation will be concealed by using sequentially numbered opaque sealed envelopes that contain group assignments determined by computer-generated random sequences.

### Blinding

Given the nature of the intervention, it is not possible to blind participants to researchers involved in providing the intervention and data collection. However, allocation to intervention or control groups will be blinded for researchers in the data set available during the data analysis. To avoid the potential contamination between two groups, we will provide the educational intervention after the typical premarital counselling.

### Statistical method

Categorical variables will be presented by frequency (%), and quantitative variables will be reported as means (SD) or medians (with 25th to 75th quartiles), as appropriate. Graphical approach, numerical indices and the Shapiro-Wilk test will be used to evaluate the normality distribution and Leven test for homogeneity of variances in quantitative variables, respectively.

Comparisons between groups at baseline will be performed using Chi-square or Fisher-exact test for categorical variables, Independent samples t-test for normally distributed quantitative variables and Mann–Whitney U test for non-normally distributed quantitative variables. To compare the outcomes between the two groups over the time, repeated measures ANOVA will be used. Data will be analyzed using the SPSS software version 22 (IBM Corp. IBM SPSS Statistics for Windows, Armonk, NY, USA). For all analyses, a *P* value < 0·05 will be considered statistically significant.

### Ethical considerations

The ethical approval of the research project was obtained from the ethics committee of National Institute for Medical Research Development (NIMAD) (IR.NIMAD.REC.1399.123). Eligible couples will be invited for an additional counselling by a health provider to ensure that they are entirely informed on the nature of the research by means of oral and written information. All the participants who agree to participate in the study, will sign a written informed consent before the randomization. All participants have the right to withdraw from the study anytime and for any reasons. The educational package will be provided to the control group as well upon their request at the end of the study. To protect against loss of confidentiality, data sheets will be stored on a password-protected computer, which can be accessed only by the members of the research team. Information on participants will be sent to the PI only as de-identified, aggregate data.

## Discussion

This research is a novel study because few studies evaluated the effect of educational interventions on fertility knowledge. In addition, to the authors’ knowledge, there is no published study that evaluated the impact of a fertility educational intervention on fertility knowledge, intention and the planned pregnancy rate using a randomized controlled trial in Iran. The focus of current study is on primary prevention and the study is designed to promote fertility knowledge and to reduce involuntary childlessness in a country with the birth rate below replacement-level. Although, it is difficult to encourage people to have more children, we can increase the knowledge of young couples in order to make informed decisions about the timing of their childbearing and assist them to achieve their fertility ideals by preventing delayed childbearing, particularly the first birth.

In this study, an educational package including videos, lectures and virtual messages will be designed, which will employ the power of social media. Unlike other high-cost interventions, this low-cost educational intervention will help couples for making informed fertility decisions. This intervention will be designed with an infertility prevention approach and childbearing motivation among young childless couples.

This study is pioneering study which uses the capabilities of social media for training and for follow up and data gathering. We also employ a multi-city sampling approach and long-term follow-up among a unique target group (about to married couples who attend compulsory premarital counselling programs). Therefore, if effective, this intervention can inform government policies to incorporate such an education to the premarital counselling courses. In this way, we hope to be able to encourage new couples to think and decide on their first birth by having all knowledge about fertility and consequences of delayed fertility.

We hypothesize that with improving fertility knowledge among new couples, the involuntary childlessness, personal and societal cost of infertility and Assisted Reproductive Technology (ART) will be decreased. The findings may also inform government policies and public education strategies aiming at supporting childbearing among young couples who postpone their first birth while they might not have any important social and economic obstacles.

## Data Availability

Not applicable, as this is a protocol manuscript.

## Abbreviations

CFKS: Cardiff Fertility Knowledge Scale
NIMAD: National Institute for Medical Research Development
ART: Assisted Reproductive Technology
STDs: Sexually Transmitted Diseases
ASRM: American Society for Reproductive Medicine
ESHRE: European Society of Human Reproduction and Embryology
MOHME: Ministry of Health and Medical Education
